# Twenty-six additional new combinations in the *Magnolia* (Magnoliaceae) of China and Vietnam

**DOI:** 10.3897/phytokeys.146.52114

**Published:** 2020-04-30

**Authors:** Christopher B. Callaghan, Siak K. Png

**Affiliations:** 1 Australian Bicentennial Arboretum, P.O. Box 88, Penshurst. NSW 2222. Australia Australian Bicentennial Arboretum Sydney Australia

**Keywords:** Magnolioideae, *
Manglietia
*, *
Michelia
*, morphological features, synonyms, *
Yulania
*

## Abstract

In accordance with the previous reduction of the remaining genera of subfamily Magnolioideae (Magnoliaceae) into the genus *Magnolia*, twenty-six new nomenclatural combinations are formally made by transferring to *Magnolia* some additional Chinese and Vietnamese taxa from the segregate genera of *Manglietia*, *Michelia* and *Yulania*. The following nine new combinations are created from *Manglietia*, namely *Magnolia admirabilis*, *M. albistaminea*, *M. guangnanica*, *M. jinggangshanensis*, *M. maguanica*, *M. pubipedunculata*, *M. pubipetala*, *M. rufisyncarpa* and *M. sinoconifera*. Also, twelve new combinations are created from *Michelia*, namely *Magnolia caloptila*, *M. caudata*, *M. fallax*, *M. gelida*, *M. hunanensis*, M. maudiae var. rubicunda, *M. multitepala*, *M. platypetala*, *M. rubriflora*, *M. septipetala*, *M. sonlaensis*, *M. xinningia*. Finally, five new combinations are created from *Yulania*, namely *Magnolia baotaina*, *M. pendula*, M. pilocarpa var. ellipticifolia, *M. puberula* and *M. urceolata*.


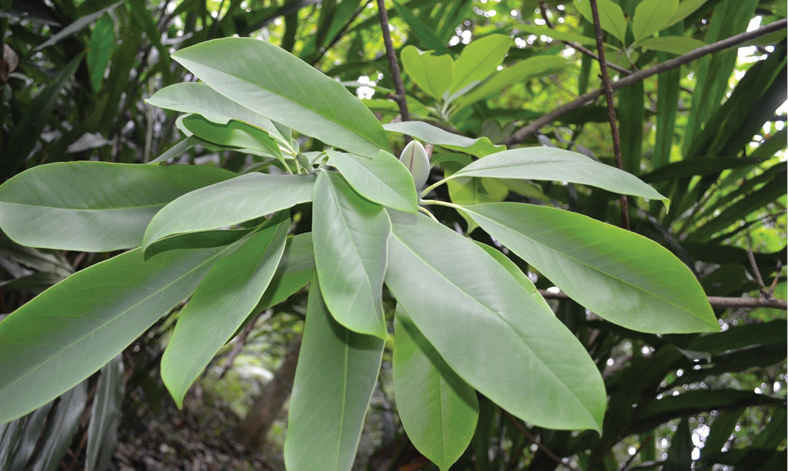


*Magnolia maguanica* (formerly *Manglietia maguanica* (photo taken by SK Png at South China BG on 21.04.2017).

## Introduction

Richard B. Figlar (2012), a past president and present scientific advisor of Magnolia Society International, provides a concise but thorough background to the complex generic history of subfamily Magnolioideae of family Magnoliaceae, starting with J.E. Dandy in the early part of the previous century. This pre-eminent British plant taxonomist, specialising in Magnoliaceae, recognised the family as consisting of 2 tribes, the Liriodendreae representing the single distinct genus *Liriodendron*, with the remainder of the family, about which Dandy (1927) acknowledges there had never been uniformity of opinion, forming the Magnolieae, comprising 9 genera, which he subsequently increased by 2.

Revisions were to follow Dandy’s death in late 1976, including the classification of the leading Chinese Magnoliaceae researcher, Liu Yu-hu (aka Law Yuh-wu). His proposed Taxonomic System of Magnoliaceae (Law 1984), republished in Magnolias of China in the year he died (Liu et al. 2004), basically added a further 4 genera to those of Dandy. Representatives of 10 of the 15 genera included in subfamily Magnolioideae in Liu’s classification occur in China.

H.P. Nooteboom, who was to succeed Dandy at the forefront of Magnoliaceae research in Europe, realised that his predecessor had been mistaken in his interpretation of certain morphological characters and thus commenced his reduction of Magnolioideae (Nooteboom 1985), to just 6 genera. Ultimately, with the advent of molecular DNA sequencing data (Azuma et al. 1999, 2000, 2001, Kim et al. 2001, Nie et al. 2008, Wang et al. 2006, Kim and Suh 2013), combined with comparative morphological research (Figlar 2000, Figlar and Nooteboom 2004), showing the remaining genera, including *Manglietia* Blume and *Michelia* Linnaeus, residing among the other sections of *Magnolia*, Figlar and Nooteboom proposed a new classification system in their 2004 paper. Their new system includes *Magnolia* at the head of a now monogeneric Magnolioideae subfamily comprising subgenus Magnolia with 8 sections and 7 subsections, subgenus Yulania with 2 sections and 6 subsections, and subgenus Gynopodium with 2 sections.

This system was not followed in the Flora of China treatment of Magnoliaceae (Xia et al. 2008), where previously recognised genera such as *Manglietia* and *Michelia* were retained, two former sections of *Magnolia* were given generic status as *Houpoea* N.H. Xia & C.Y. Wu and *Oyama* (Nakai) N.H. Xia & C.Y. Wu, and former genera, such as *Lirianthe* Spach and *Yulania* Spach of 1839 were reinstated. Since then, authors describing new species from China have followed this classification, a few examples being *Manglietia pubipedunculata* Q.W. Zeng & X.M. Hu (Hu et al. 2019), *Michelia caudata* M.X. Wu, X.H. Wu & G.Y. Li (Wu et al. 2015) and *Yulania dabieshanensis* T.B. Zhao, Z.X. Chen & H.T. Dai (Dai et al. 2012).

However, Figlar and Nooteboom’s (2004) classification system is now widely accepted by the scientific community, with many authors following this broad view of *Magnolia*, such as Arroyo et al. (2013), Ninh et al. (2020), Pérez et al. (2016) and Zou et al. (2020).

Figlar (2012) advised against the alternative classification system now operating:

*In a one genus system only Manglietia, Michelia and 3 minor genera require new names in Magnolia. In a 13 genera system, it would be necessary to dismantle the largest and most well-known genus, Magnolia, and rename the constituents into 10 new genera. That would be enormously destructive to the long-established Magnolia-centric nomenclature and literature, causing unnecessary and undesirable consequences to science, conservation and horticulture*.

With this in mind, 26 new combinations are created here, representing nine species of *Manglietia*, eleven species and one variety of *Michelia*, plus four species and one variety of *Yulania*. Most of these taxa were named and described over the past decade, but include some older previously synonymised, now reinstated taxa, that are herein transferred to *Magnolia*, as will be numerous other taxa in a sequel to this paper (Callaghan and Png 2019a, 2020).

## Materials and methods

The new combinations proposed in this paper are made in compliance with the rules and recommendations of the 2018 International Code of Nomenclature for algae, fungi and plants (ICN), known as The Shenzhen Code (Turland et al. 2018), in particular ICN Article 41 and Recommendation 41A in respect of new combinations.

Where available, digital images of type specimens of newly named taxa posted to the internet at the websites of various herbaria have been sighted and these are indicated in the text by ‘online image!’ appearing after the herbarium acronyms whose representative names are listed in the appendix following the references. Additional sighted specimens are indicated by ‘!’ after the herbarium acronym.

Consultation of the relevant literature was made to determine whether a number of taxa previously determined as synonyms of earlier named taxa were, in fact, genuine independent species or varieties as they had been originally described. Differences in numerous morphological features, natural distributions and/or elevations and where appropriate, the incompatible phenology of flowering and/or fruiting periods, are tabulated and referred to in the notes under the relevant taxa to fully substantiate their independent status.

Floras and other literature dealing with the Magnoliaceae of China that have been consulted during this study are cited in the text, with some of the more important sources of information including a number of papers by Dandy (1928 a–c, 1930), The Magnoliaceae of China (Chen and Nooteboom 1993), Magnoliaceae in Flora Reipublicae Popularis Sinicae (Law et al. 1996), Magnolias of China (Liu et al. 2004), Magnoliaceae in Flora of China Vol. 7 (Xia et al. 2008), A Taxonomic Revision of the Magnoliaceae from China (Sima 2011) and the recent Ex Situ Cultivated Flora of China : Magnoliaceae (Yang et al. 2016), which documents the diversity of Magnoliaceae plants in Chinese botanical gardens.

The Biodiversity Heritage Library website (https://www.biodiversitylibrary.org) proved indispensable in accessing a number of articles on earlier-named Magnoliaceae dating back to the early nineteenth century and beyond. A good proportion of the numerous relevant scientific and mainstream literature consulted during this research is internet accessible via the links included with the references. The links included in the 2019 unpublished version of this paper were rechecked to confirm their current accessibility.

## Results

In accordance with the previous reduction of the remaining genera of subfamily Magnolioideae (Magnoliaceae) into the genus *Magnolia*, twenty-six new nomenclatural combinations are formally made by transferring to *Magnolia* some additional Chinese and Vietnamese taxa from the segregate genera of *Manglietia*, *Michelia* and *Yulania* that were described during the past decade and occasionally earlier, plus a few formerly synonymised, now reinstated taxa.

The following nine new combinations are created from *Manglietia*, namely *Magnolia admirabilis* (Y.H. Law & R.Z. Zhou ex L. Fu, Q.W. Zeng & X.M. Hu) C.B. Callaghan & S.K. Png, *M. albistaminea* (Y.W. Law, R.Z. Zhou & S.X. Qin) C.B. Callaghan & S.K. Png, *M. guangnanica* (D.X. Li & R.Z. Zhou ex X.M. Hu, Q.W. Zeng & L. Fu) C.B. Callaghan & S.K. Png, *M. jinggangshanensis* (R.L. Liu & Z.X. Zhang) C.B. Callaghan & S.K. Png, *M. maguanica* (H.T. Chang & B.L.Chen) C.B. Callaghan & S.K. Png, *M. pubipedunculata* (Q.W. Zeng & X.M. Hu) C.B. Callaghan & S.K. Png, *M. pubipetala* (Q.W. Zeng) C.B. Callaghan & S.K. Png, *M. rufisyncarpa* (Y.W. Law, R.Z. Zhou & F.G. Wang) C.B. Callaghan & S.K. Png and *M. sinoconifera* (F.N. Wei) C.B. Callaghan & S.K. Png.

Also, twelve new combinations are created from *Michelia*, namely *Magnolia caloptila* (Y.W. Law & Y.F. Wu) C.B. Callaghan & S.K. Png, *M. caudata* (M.X. Wu, X.H. Wu & G.Y. Li) C.B. Callaghan & S.K. Png, *M. fallax* (Dandy) C.B. Callaghan & S.K. Png, *M. gelida* (T.B. Zhao, Z.X. Chen & D.L. Fu) C.B. Callaghan & S.K. Png, *M. hunanensis* (C.L. Peng & L.H. Yan) C.B. Callaghan & S.K. Png, M. maudiae var. rubicunda (T.P. Yi & J.C. Fan) C.B. Callaghan & S.K. Png, *M. multitepala* (R.Z. Zhou & S.G. Jian) C.B. Callaghan & S.K. Png, *M. platypetala* (Hand-Mazz.) C.B. Callaghan & S.K. Png, *M. rubriflora* (Y.W. Law & R.Z. Zhou ex F.G. Wang, Q.W. Zeng, R.Z. Zhou & F.W. Xing) C.B. Callaghan & S.K. Png, *M. septipetala* (Z.L. Nong) C.B. Callaghan & S.K.Png, *M. sonlaensis* (Q.N. Vu) C.B. Callaghan & S.K. Png and *M. xinningia* (Y.W. Law & R.Z. Zhou ex Q.X. Ma, Q.W. Zeng, R.Z. Zhou & F.W. Xing) C.B. Callaghan & S.K. Png.

Finally, five new combinations are created from *Yulania*, namely *Magnolia baotaina* (D.L. Fu, Q. Zhang & M. Xu) C.B. Callaghan & S.K. Png, *M. pendula* (D.L. Fu) C.B. Callaghan & S.K. Png, M. pilocarpa var. ellipticifolia (Z.Z. Zhao & Z.W. Xie) C.B. Callaghan & S.K. Png, *M. puberula* (D.L. Fu) C.B. Callaghan & S.K. Png and *M. urceolata* (D.L. Fu, B.H. Xiong & X. Chen) C.B. Callaghan & S.K. Png.

## Discussion

The transfer of the above twenty-six taxa to *Magnolia* is necessary following the present near universal acceptance by the scientific community and horticultural industry that the Magnolioideae is one of two monogeneric subfamilies within Magnoliaceae and the fact that the majority of resulting new combinations and names arising from the relegation of *Manglietia* and *Michelia* into *Magnolia* have previously been made by various authors such as Figlar (2000) for the majority of the *Michelia* species, with Sima (2001) transferring some additional *Michelia* species, Kumar (2006) transferring the majority of *Manglieta* species, Nooteboom transferring a number of species from both the previous genera plus *Yulania* in Flora of China Vol. 7 (Xia et al. 2008: 49–50) and most recently Callaghan and Png (2013) transferring species from these three genera that were mainly described and named subsequent to the publication of Flora of China.

## Conclusions

To maintain these twenty-six predominantly recently described taxa in limbo in segregate genera will contribute to further instability and inevitable confusion in the scientific and popular literature, as well as within the botanical world and the horticultural industry, which has resulted from having two diverse systems operating simultaneously.

The authors would like to take this opportunity to suggest that to further substantiate their now reaffirmed species or varietal status, comparative DNA barcoding (Caddy-Retalic and Lowe 2012), should be undertaken of these and other taxa, often with small remnant populations and/or disjunct geographic distributions, that have been previously subsumed in synonymy under earlier-named species having much larger populations of widespread occurrence. As a result of becoming virtual non-entities, this can be detrimental to their conservation and ultimate survival in nature. Consequently their potential benefits to mankind, such as the medicinal properties that some Magnoliaceae species are known to possess, including present and prospective production of anti-cancer drugs and treatments (He et al. 2017, Huang et al. 2017, Lu et al. 2017, Ma et al. 2020, Prasad and Katiyar 2018, Zhang et al. 2020), are never assessed or realised.

## Taxonomic section

### Magnolia admirabilis

Taxon classificationPlantaeMagnolialesMagnoliaceae

(Y.H. Law & R.Z. Zhou ex L. Fu, Q.W. Zeng & X.M. Hu) C.B. Callaghan & S.K. Png comb. nov.

39B777BF-CF62-5591-B894-9D8115202518

urn:lsid:ipni.org:names:77209515-1

#### Basionym.

*Manglietia admirabilis* Y.H. Law & R.Z. Zhou ex L. Fu, Q.W. Zeng & X.M. Hu, Novon 23(1): 37, fig. 1 (2014).

#### Chinese name.

奇异木莲 meaning “distinctive Manglietia”

#### Type.

CHINA. Yunnan Province: Maguan County, Gulinqing, Chuntianping, ca. 1300 m, limestone montane evergreen broad-leaved forests, 12 May 1986, *Zhou Ren-zhang 98* (holotype: IBSC n.v.). Guangdong Province: Guangzhou, Magnolia Garden of South China Botanical Garden, ca. 50 m, 3 May 2011, *Lin Fu 20110503* (paratype: IBSC n.v.)

#### Note.

There is no data or images held at IBSC for the holotype (Huang Xiangxu, pers. comm., July 2019).

### Magnolia albistaminea

Taxon classificationPlantaeMagnolialesMagnoliaceae

(Y.W. Law, R.Z. Zhou & X.S. Qin) C.B. Callaghan & S.K. Png comb. nov.

7A5094BC-C6E0-556C-9CC6-76829D13BDFA

urn:lsid:ipni.org:names:77209516-1

#### Basionym.

*Manglietia albistaminea* Y.W. Law, R.Z. Zhou & X.S. Qin. In: X.S. Qin et al., Novon 16: 260, fig. 1 (2006).

#### Chinese name.

白蕊木莲 meaning “white-stamened manglietia”

#### Type.

CHINA. Guangdong Province: South China Botanical Garden, Guangzhou (collected from plant introduced in 1982 from Mt. Jianfengling, Ledong County, Hainan), 10 May 2001, *R.Z. Zhou 130* (holotype: IBSC n.v.; isotype: MO n.v.). Same locality (collected from plant introduced as above) 23 April 1999, *R.Z. Zhou 9916* and *R.Z. Zhou 0136* (paratypes: IBSC n.v.).

Manglietia fordiana Oliv. var. hainanensis (Dandy) N.H. Xia. In: Xia et al. (2008: 58), p.p. quoad syn. *Manglietia albistaminea* Y.W. Law et al.

*Manglietia fordiana* Oliv. In: Sima and Lu (2009: 23) and Sima (2011: 88), both p.p. quoad syn. *Manglietia albistaminea* Y.W. Law et al.

#### Note.

There are no data or images held at IBSC for the holotype (Huang Xiangxu, pers. comm., July 2019), nor could the isotype be located at MO (Jim Solomon, pers. comm., July 2019).

### Magnolia baotaina

Taxon classificationPlantaeMagnolialesMagnoliaceae

(D.L. Fu, Q. Zhang & M. Xu) C.B. Callaghan & S.K. Png comb. nov.

234BA9EF-EAC8-50A1-9E43-35AD768A6C6E

urn:lsid:ipni.org:names:77209518-1

#### Basionym.

*Yulania baotaina* D.L. Fu, Q. Zhang & M. Xu. In: D.L. Fu et al., Amer. J. Agric. and Forest. 7(5): 231–232, fig. 1 (2019c).

#### Chinese name.

宝台山玉兰 meaning “Mount Baotai yulania”

#### Type.

CHINA. Yunnan Province: Yongping County, Mount Baotai, 2600 m, 12 March 2017, *D.L. Fu 2017031201* (holotype: CAF n.v.). Same locality, 9 September 2017, *D.L. Fu 2017093001* (paratype: CAF n.v.).

#### Note.

The type specimens of *Yulania baotaina* cannot be located at the Beijing herbarium of CAF (Wang Hongbin, pers. comm., March 2020).

### Magnolia caloptila

Taxon classificationPlantaeMagnolialesMagnoliaceae

(Y.W. Law & Y.F. Wu) C.B. Callaghan & S.K. Png comb. nov.

738615A7-117F-5C82-83A3-AC9881503ACC

urn:lsid:ipni.org:names:77209519-1

#### Basionym.

*Michelia caloptila* Y.W. Law & Y.F. Wu. In: Bull. Bot. Res., Harbin 4(2): 152, 154: fig. *s.n.* (1984).

#### Chinese name.

美毛含笑 meaning “beautiful-haired michelia”

#### Type.

CHINA. Jiangxi Province: Zixi County, Nangang, Matoushan, 450 m, in woods, 17 September 1980, *Jiangxi gong-da linxue-xi (JXAU) 80069* (holotype: IBSC! + online image!; isotypes: LBG online images!).

Digital images of type specimens below accessed 19 March 2019:

holotype [IBSC: 0003281]: http://www.docin.com/p-1050989203.html (Sima 2011: 316, photo 2-58).

isotype [LBG: 00004082]: http://www.cvh.ac.cn/spm/LBG/00004082

isotype [LBG: 00004123]: http://www.cvh.ac.cn/spm/LBG/00004123

*Michelia fujianensis* Q.F. Zheng. In: Xia and Deng (2002: 130) and Xia et al. (2008: 83), both p.p. quoad syn. *Michelia caloptila* Y.W. Law & Y.F. Wu.

*Michelia caloptila* Y.W. Law & Y.F. Wu. In: Sima (2011: 234), p.p. excl. syns. *Michelia concinna* H. Jiang & E.D. Liu and *Michelia septipetala* Z.L. Nong.

#### Note 1.

*Michelia caloptila* Y.W. Law & Y.F. Wu was listed as a dubious species in Chen and Nooteboom (1993: 1088), in which it was noted that specimens had not been seen. It was subsequently reduced to a synonym of *Michelia fujianensis* as noted above. It is recognised as a genuine species by Law et al. (1996: 189), Liu et al. (2004: 228), Deng and Yang (2015: 167), Yang et al. (2016: 237) and Sima (2011: 234), wherein *M. caloptila* is in Michelia subsection Micheliopsis, series *Micheliopsis* and *M. fujianensis* is in Michelia subsection Velutinae. Differences between the abaxial indumentum of the 9–16 cm long leaves of *M. caloptila* and of the 6–11 cm long leaves of *M. fujianensis* are illustrated in Plate 3-2E (*M. caloptila*) and Plate 3-3E (*M. fujianensis*) of Sima (2011: 325; 326). Further substantiation of the specific status of *M. caloptila* is evident from a comparison of its morphological features with those of *M. fujianensis*, as shown in Table 1 on the following page.

**Table 1.** Differentiating features of the species *Michelia caloptila* and *Michelia fujianensis*.

**Plant feature**	***Michelia caloptila* Y.W. Law & Y.F. Wu**	***Michelia fujianensis* Q.F. Zheng**
maximum dimensions	to 15 m × 30 cm dbh^†^	to 16 m × 100 cm dbh^¶^
bark colour	grey	greyish-brown (greyish-white^¶^)
indumentum of branchlets	brown tomentose	densely cinnamon tomentose
indumentum of buds	brown tomentose	densely cinnamon tomentose
leaf shape	narrowly elliptic or elliptic	oblong or narrowly obovate-elliptic
leaf dimensions	9–16 × 2.5–5 cm	6–11 × 2.5–4 cm
leaf apex	acuminate or caudate-acuminate	acute
leaf indumentum adaxially	entirely glabrous	densely short-tomentose at midrib
leaf indumentum abaxially	minutely brown tomentose	densely ferrugineus or brownish-yellow appressed sericeous
secondary lateral leaf veins	7–12 pairs	8–9 pairs (not 9–15^§,¶^)
petiole length and indumentum	5–10 mm, brown tomentose	10–15 mm, densely cinnamon tomentose
tepal number	6–9^‡^	15–16 (12–17^¶^)
gynophore in fruit	ca. 20 mm long	2–2.5 mm long
fruit aggregate length	4–10 cm	2–3 cm
mature carpels	broadly ovate or suborbicular, 1–1.8 cm long with 1–4 seeds	obovoid, 1.5–2 cm × ca. 1.2 cm with 1 seed
fruiting period	September^†^	October–November^¶^

The differentiating features of *Michelia caloptila* are cited from Law and Wu (1984) to whom the flower was unknown, Liu et al. (2004: 228)^†^ and Yang et al. (2016: 237)^‡^, with those of *M. fujianensis* from Zheng (1981), supplemented by Law et al. (1996: 189)^§^ and Liu et al. (2004: 260)^¶^.

#### Note 2.

As a consequence of the above substantiation of the species status of *Michelia caloptila*, plus the past reduction to *Magnolia* of the remaining genera of subfamily Magnolioideae, *Michelia caloptila* is here transferred to *Magnolia*.

### Magnolia caudata

Taxon classificationPlantaeMagnolialesMagnoliaceae

(M.X. Wu, X.H. Wu & G.Y. Li) C.B. Callaghan & S.K. Png comb. nov.

05B989BC-5C2D-5392-9ACC-EC847CB35336

urn:lsid:ipni.org:names:77209521-1

#### Basionym.

*Michelia caudata* M.X. Wu, X.H. Wu & G.Y. Li. In: X.H. Wu et al., Acta Bot. Bor-Occid. Sin. 35(5): 1058, fig. 1 (2015).

#### Chinese name.

尾叶含笑 meaning “caudate-lobed michelia”, referring to shape of leaf apex.

#### Type.

CHINA. Zhejiang Province: Qingyuan County, Songyuan town, Jiaokeng village, Guanmenao Conservation Area, in evergreen broad-leaved forests, ravines, 460 m, 12 April 2010, *Ye Qing-jiao & Wu Xia-hua 1096* (holotype: ZJFC n.v.). Zhejiang Province: Qingyuan County, Songyuan town, Jiaokeng village, 460 m, 26 September 2010, *Ma Dan-dan, Li Gen-you, Wu Ming-xiang QY20100922* (paratype: ZJFC n.v.).

### Magnolia fallax

Taxon classificationPlantaeMagnolialesMagnoliaceae

(Dandy) C.B. Callaghan & S.K. Png comb. nov.

9F7555EE-8C9E-522C-A75C-20539022D6ED

urn:lsid:ipni.org:names:77209522-1

#### Basionym.

*Michelia fallax* Dandy. In: Notes, Roy. Bot. Gard. Edinburgh 16(77): 130 (1928c).

**Chinese names**: 灰绒含笑 meaning “grey-velvet michelia”, referring to the grey indumentum covering branchlets, buds, etc. Also: 大叶含笑 meaning “large-leaved michelia”

#### Type.

CHINA. Hunan Province: near Wukang-chow (=Wugang), Yunshan, ca. 950 m, in lofty shady forests, 12 July 1918, *Handel-Mazzetti 12281* p.p. quoad fruiting specimen (holotype: WU online image!; isotypes: A online image!, K online image!).

Digital images of holotype and isotype specimens below accessed 19 March 2019:

holotype [WU: 0039581]: http://herbarium.univie.ac.at/database/detail.php?ID=70940

isotype [A: 00039058]: https://s3.amazonaws.com/huhwebimages/6C9726D2157D489/type/full/39058.jpg

isotype [K: K000681458]: http://apps.kew.org/herbcat/getImage.do?imageBarcode=K000681458

*Michelia cavaleriei* Finet & Gagnep. In: Chen and Nooteboom (1993: 1058), Frodin and Govaerts (1996: 55), Wu and Chen (2006: 56), Sima and Lu (2009: 50), Sima (2011: 214), Deng and Yang (2015: 148), each p.p. quoad syn. *Michelia fallax* Dandy.

Michelia cavaleriei Finet & Gagnep. var. cavaleriei. In: Xia et al. (2008: 84), p.p. quoad syn. *Michelia fallax* Dandy.

#### Note 1.

James E. Dandy (1928c: 130), provides background information concerning the division of the fruiting and flowering collections made under number *12281* by Handel-Mazzetti on 12 July 1918 and by his servant Wang Te-hui in April 1919 respectively. From his study of these collections, Dandy came to the realisation that they represented two distinct species, retaining Handel-Mazzetti’s name *Michelia platypetala* for Wang’s flowering material and publishing the name *Michelia fallax* for Handel-Mazzetti’s fruiting material.

#### Note 2.

An undated identification label in the name of J.E. Dandy, affixed to the Kew Herbarium isotype specimen of *M. fallax*, indicates his subsequent determination of it as *M. cavaleriei* Finet & Gagnep. This specimen and the other above seen type specimens are all ca. 20 cm in length and 6 cm wide, roughly only about two-thirds of Dandy’s original description of the leaves of *M. fallax* being “usque ad ca. 30 cm longa et 8.5 cm lata” (up to about 30 cm long and 8.5 cm wide). Dandy’s dimensions are not a misprint, since there are a number of *M. fallax* specimens of different provenances (and provinces) posted to the Chinese Virtual Herbarium (CVH) website with leaves approaching this size, which is alluded to in one of this species two Chinese names translating as “large-leaved michelia”. The above noted dimensions must be presumed to be those of the other specimen noted in Dandy’s description, the undated specimen *Dalziels.n.*, collected at about 900 m near Thai-yong, 97 km west of Swatow (Shantou) on Guangdong’s northeastern coast, sometime between 1895 and 1902 (this specimen was not located for the current research).

#### Note 3.

Subsequent to Dandy, *M. fallax* has been listed as a synonym of *M. cavaleriei* and of M. cavaleriei var. cavaleriei by the authors cited in the section preceding Note 1. However, the present authors consider that while these two species are superficially similar in the shape of their leaves, that the known comparative features recorded in Table 2 below distinguish *Michelia fallax* as an independent species. Also, it does not key out with the original validating descriptions for *Michelia hunanensis* or *M. xinningia* with which it shares synonymy under M. cavaleriei var. cavaleriei in Flora of China. Therefore, consistent with the past reduction to *Magnolia* of the remaining segregate genera of subfamily Magnolioideae, *Michelia fallax* is here transferred to *Magnolia*.

**Table 2.** Differentiating features of the species *Michelia fallax* and *Michelia cavaleriei*.

**Plant feature**	***Michelia fallax* Dandy**	***Michelia cavaleriei* Finet & Gagnep.**
indumentum of branchlets	appressed grey tomentose, becoming tawny near apex	silver-grey or rufous appressed pilose^§^
indumentum of buds	appressed shiny grey tomentose	silver-grey or rufous appressed pilose^§^
leaf shape	elliptic-oblong, oblong or narrowly oblong	narrowly oblanceolate-oblong or narrowly oblong^§^
leaf apex	acuminate or subacuminate	acuminate or short-acute^§^
leaf base	obtuse or sub-rounded	cuneate or broadly cuneate^§^
leaf dimensions	up to ca. 30 × 8.5 cm (ca. 29 × 9 cm^†^)	10–20 × 3.5–6.5 cm^§^
leaf indumentum abaxially	short appressed grey pubescent	glaucous, silver-grey or rufous appressed pilose when young^§^
secondary lateral leaf veins	ca. 14–16 pairs	11–15 pairs^‡^
petiole length and indumentum	ca. 2.5 cm, initially appressed grey or yellow-brown tomentose, later glabrescent	2 cm, puberulus (0.7–1.5 cm^#^ silver-grey or rufous appressed pilose^§^)
gynoecium indumentum	appressed grey tomentellous	glabrous except for few bristly hairs towards apex of carpels^††^
number of ovules	ca. 10	2
fruiting peduncle indumentum	appressed glossy grey or yellowish-brown tomentose	silver-grey or rufous appressed pilose^§^
fruit aggregate length	10–12 cm	5–10 cm^§^
mature carpels length	up to ca. 2.5 cm	1.5–2 cm^‡^
fruiting period	July	September–October^‡,§^

The differentiating features of *Michelia fallax*, whose flower is unknown, are cited from Dandy (1928c) and CVH (2017)^†^; those of *M. cavaleriei* from Finet & Gagnepain (1906), Law et al. (1996: 184)^‡^, Liu et al. (2004: 229)^§^ and Yang et al. (2016: 239)^#^, plus Dandy (1928c: 130)^††^.

### Magnolia gelida

Taxon classificationPlantaeMagnolialesMagnoliaceae

(T.B. Zhao, Z.X. Chen & D.L. Fu) C.B. Callaghan & S.K. Png comb. nov.

6157C94A-B213-55FC-AEC5-3FD3577AB80A

urn:lsid:ipni.org:names:77209524-1

#### Basionym.

*Michelia gelida* T.B. Zhao, Z.X. Chen & D.L. Fu. In: Y.F. Hu et al., Advances Orn. Hort. China 2013: 39–40, fig. 1 (2013).

#### Chinese name.

耐冬含笑 meaning “winter resisting michelia”

#### Type.

CHINA. Henan Province: Jinling County, Changge city, cultivated (native to Zhejiang Province: Fuyang County), 24 March 2010, *Zhao Tian-bang, Fu Da-li et al. 201003245* (holotype: HEAC, fol, fl. n.v.)

### Magnolia guangnanica

Taxon classificationPlantaeMagnolialesMagnoliaceae

(D.X. Li & R.Z. Zhou ex X.M. Hu, Q.W. Zeng & L. Fu) C.B. Callaghan & S.K. Png comb. nov.

0412A48E-1132-508C-BD23-9B64F81CAB78

urn:lsid:ipni.org:names:77209525-1

#### Basionym.

*Manglietia guangnanica* D.X. Li & R.Z. Zhou ex X.M. Hu, Q.W. Zeng & L. Fu, Novon 23(2): 172, figs. 1, 2 (2014).

#### Chinese name.

广南木莲 meaning “Guangnan manglietia”

#### Type.

CHINA. Yunnan Province: Guangnan County, Heizhiguo town and village, Mt. Gulu, in limestone montane evergreen broad-leaved forest, 1710 m, 17 October 1993, *Zhou Ren-zhang & Zeng Qing-wen 93049* (holotype and isotype: IBSC n.v.). Same locality, 12 May 1992, *D.X. Li & Z.Q. Ouyang 920512* (paratype: MO n.v.). Same locality 16 April 2003, *R.Z. Zhou 03046* (paratype: IBSC n.v.). Yunnan Province: Guangnan County, Mount Houshan, near Zhujie village of Zhujie town, 1600 m, 4 October 1993, *R.Z. Zhou 9304* (paratype: IBSC!). Yunnan Province: Kunming Botanical Garden, cultivated, 1 May 2010, *X.M. Hu & Q.W. Zeng 00166* (paratype: IBSC n.v.).

#### Note.

The holotype and isotype specimens of *Manglietia guangnanica* could not be found by herbarium staff at IBSC, nor could the paratype specimen at MO be located (Jim Solomon, pers. comm., July 2019). However, the paratype that was received from IBSC, *R.Z. Zhou* (*Zhou Ren-zhang*) *9304* collected at 1600 m, inexplicably has the locality and collection date as for the holotype / isotype above and not Mount Houshan on the 4 October 1993 as is noted in the 2014 paper for this paratype.

### Magnolia hunanensis

Taxon classificationPlantaeMagnolialesMagnoliaceae

(C.L. Peng & L.H. Yan) C.B. Callaghan & S.K. Png comb. nov.

25A0FEB1-20D1-5504-B86E-7D87987D846E

urn:lsid:ipni.org:names:77209526-1

#### Basionym.

*Michelia hunanensis* C.L. Peng & L.H. Yan. In: C.L. Peng et al., J. Hunan Forest. Tech. Coll. 1995(1): 15 (1995).

#### Chinese name.

湖南含笑 meaning “Hunan michelia”

#### Type.

CHINA. Hunan Province: Xinning County, without elevation or collection date, *L.H. Yan & C.L. Peng 93018* (holotype: HFBG n.v.; isotype: HFTC n.v.).

Magnolia maudiae (Dunn) Figlar var. hunanensis (C.L. Peng & L.H. Yan) Sima (2001: 33).

*Michelia cavaleriei* Finet & Gagnep. In: Xia & Deng (2002: 132) and Sima (2011: 214), both p.p. quoad syn. *Michelia hunanensis* C.L. Peng & L.H. Yan—Sima & Lu (2009: 50), p.p. quoad syns. *Michelia hunanensis* C.L. Peng & L.H. Yan and Magnolia maudiae (Dunn) Figlar var. hunanensis (C.L. Peng & L.H. Yan) Sima.

Michelia cavaleriei Finet & Gagnep. var. cavaleriei. In: Xia et al. (2008: 84), p.p. quoad syn. *Michelia hunanensis* C.L. Peng & L.H. Yan.

#### Note 1.

The holotype specimen was irretrievably damaged during repeated relocations of the HFBG herbarium (Yan Lihong, pers. comm.). Photographs were sent in its place.

**Note 2.** The numerous known differentiating features compiled in Table 3 below confirm *Michelia hunanensis* as an independent species and not a variety of *Magnolia maudiae*, nor a synonym of Michelia cavaleriei var. cavaleriei as noted above.

**Table 3.** Differentiating features of *Michelia hunanensis*, *M. maudiae* and *M. cavaleriei*.

**Plant feature**	***Michelia hunanensis* C.L. Peng & L.H. Yan**	***Michelia maudiae* Dunn**	***Michelia cavaleriei* Finet & Gagnep.**
maximum height	20 m	31 m^§^	10 m^¶¶^
indumentum of buds	greyish-pilose	glabrous (covered with white powder^††^)	silver-grey or rufous appressed pilose^¶¶^
leaf shape	oblong or broadly oblong	oblong-elliptic or occasionally ovate-elliptic^††^	narrowly oblanceolate-oblong or narrowly oblong^¶¶^
leaf dimensions	13–33 × 6–9 cm	7–18 × 3.5–8.5 cm^††^	10–20 × 3.5–6.5 cm^¶¶^
leaf apex	cuspidate	obtuse acuminate (occasionally long-acuminate^†^)	acuminate or short-acute^¶¶^
leaf base	rounded or obtuse	acute or cuneate	cuneate or broadly cuneate^¶¶^
undersides of leaves	greyish pubescent	glabrous, as is the entire plant^††^, except for the silky grey pubescent stamens^#^	glaucous and silver-grey or rufous appressed pilose when young^¶¶^)
lateral leaf veins	8–14 pairs	8–12 pairs	11–15 pairs^§§^
petiole length and indumentum	2–3.5 cm, pilose	2.5–3 cm, glabrous (1–3 cm^‡‡^)	2 cm, puberulus (0.7–1.5 cm)^##^, silver-grey or rufous appressed pilose^¶¶^
tepal number	9	9–11^‡‡^	10–12^##^
tepal shape and size (outer 3)	obovate, 6–7 cm long (width not specified)	obovate, 5–7 × 3.5–4 cm^††^	obovate-elliptic (2.5–4 cm long^¶¶^)
tepal shape and size (inner 3)	obovate-lanceolate, 4–4.8 × 1.2–1.4 cm	obovate, elliptic to broadly spathulate, 4.5–5 × 1.8–2.5 cm^†^	obovate-elliptic, 2.5 × 1.5 cm
length of stamens	ca. 1cm	1.5–2.2 cm^¶^	1.2–1.4 cm^§§^
gynoecium length, shape and indumentum	1 cm, cylindric, pubescent	1.5–1.8 cm (1.0–1.3 cm, subcylindric^†^), glabrous	1 cm, narrowly ovate, with a few hairs only near the carpel apex
gynophore length	5–8 mm	ca. 10 mm	ca. 4 mm^§§^
fruit aggregate length	8–17 cm	10–12(–14) cm^†^	5–10 cm^¶¶^
flowering period	March–April	January–March^††^	March^§§, ¶¶^
fruiting period	August–September	October–November^††^	September–October^§§,¶¶^

The distinguishing features of *Michelia hunanensis* are cited from Peng et al. (1995). Those of *M. maudiae* are cited from Dunn (1908), supplemented by Chen and Nooteboom (1993:1072)^†^, Deng and Yang (2015: 157)^§^, Law et al. (1996: 179)^¶^, Lee (1935: 487)^#^, Liu et al. (2004: 290)^††^ and Yang et al. (2016: 295)^‡‡^, with those of *M. cavaleriei* from Finet and Gagnepain (1906), supplemented by Law et al. (1996: 184)^§§^, Liu et al. (2004: 229)^¶¶^ and Yang et al. (2016: 239)^##^.

### Magnolia jinggangshanensis

Taxon classificationPlantaeMagnolialesMagnoliaceae

(R.L. Liu & Z.X. Zhang) C.B. Callaghan & S.K. Png comb. nov.

81146D12-2B36-5A67-B15D-EC1BB00205F5

urn:lsid:ipni.org:names:77209527-1

#### Basionym.

*Manglietia jinggangshanensis* R.L. Liu & Z.X. Zhang. In: Fedd. Repert. 130(3): 289, 290 fig. 1, 291 fig. 2 (2019)

#### Chinese name.

井冈山木莲 meaning “Jinggangshan manglietia”

#### Type.

CHINA. Jiangxi Province: Jinggangshan, in evergreen forest, 980 m, 8 May 2001 (fl.), *R.L. Liu 20010012* (holotype: BJFC!; isotypes: PE n.v., K n.v.)

#### Note.

The isotypes at PE and K could not be located (Xiaohua Jin, PE, Beijing, pers. comm., July 2019 and Clare Drinkell, assistant curator, Kew, pers. comm., July 2019).

### Magnolia maguanica

Taxon classificationPlantaeMagnolialesMagnoliaceae

(Chang & B.L. Chen) C.B. Callaghan & S.K. Png comb. nov.

0CA31536-5489-55A3-9F1A-B8874ED12525

urn:lsid:ipni.org:names:77209528-1

#### Basionym.

*Manglietia maguanica* Chang & B.L. Chen. In: B.L. Chen, Acta Sci. Nat. Univ. Sunyatseni 1988(1): 109 (1988).

#### Chinese name.

马关木莲 meaning “Maguan manglietia”

#### Type.

CHINA. Yunnan Province: Maguan County, Bazhai, near Xiaoshan, in woods, ca. 1800 m, 7 October 1986, *B.L. Chen & Y.H. Su 86s-053* (holotype: SYS! + online image!; isotype: L online image!).

Digital image of specimens below accessed 19 March 2019:

holotype (SYS): http://www.docin.com/p-1050989203.html (Sima 2011: 312, photo 2-42).

isotype [L: L0204985]: http://medialib.naturalis.nl/file/id/L0204985_MLN/format/large?fpi=1

*Manglietia insignis* (Wall.) Blume. In: Chen and Nooteboom (1993: 1044), Frodin and Govaerts (1996: 52), J. Li (1997: 132), Wu and Chen (2006: 10), and Xia et al. (2008: 56), each p.p. quoad syn. *Manglietia maguanica* Chang & B.L. Chen.

*Magnolia insignis* Wall. In: Khuraijam and Goel (2015: 109), p.p. quoad syn. *Manglietia maguanica* Chang & B.L. Chen.

#### Note.

*Manglietia maguanica* is listed as a synonym of *M. insignis* in Chen & Nooteboom (1993) and subsequently by the authors noted above. However, both are recognised as independent species in the majority of the more recent Chinese publications, including Liu et al. (2004: 164, 156), Xing et al. (2009: 198, 196), Sima and Lu (2009), Sima (2011: 98, 102), Deng and Yang (2015: 48, 54) and Yang et al. (2016: 192, 181).

### Magnolia maudiae

Taxon classificationPlantaeMagnolialesMagnoliaceae

Dunn (Figlar) var. rubicunda (T.P. Yi & J.C. Fan) C.B. Callaghan & S.K. Png comb. nov.

F64F0FF6-5548-5858-AF59-384F87CA6D14

urn:lsid:ipni.org:names:77209530-1

#### Basionym.

Michelia maudiae Dunn var. rubricunda T.P. Yi & J.C. Fan. In: J.C. Fan et al., J. Sichuan Forest. Sci. Tech. 30(4): 68, plate 1 (2009).

#### Chinese name.

红花深山含笑 meaning “red-flowered deep mountains michelia”

#### Type.

CHINA. Sichuan Province: Dujiangyan, cultivated at the Arboretum of Sichuan Agricultural University, 22 February 2009, *T.P. Yi 09001* (holotype: SAUT=SIFS, fl. n.v.). Other specimens recorded: same locality, 13 September 2008, *T.P. Yi 08005* (SAUT=SIFS, fr. n.v.). Sichuan Province: Dujiangyan Juyuan Nursery, 20 August 2008, *T.P. Yi 08004* (SAUT=SIFS, fr. n.v.). [Introduced from Tongdao County, Hunan Province].

### Magnolia multitepala

Taxon classificationPlantaeMagnolialesMagnoliaceae

(R.Z. Zhou & S.G. Jian) C.B. Callaghan & S.K. Png comb. nov.

FB96AC13-3907-5137-84B6-B7551B076C8D

urn:lsid:ipni.org:names:77209531-1

#### Basionym.

*Michelia multitepala* R.Z. Zhou & S.G. Jian. In: S.G. Jian et al., Ann. Bot. Fenn. 44: 65, fig. 1 (2007).

#### Chinese name.

多瓣含笑 meaning “multi-tepalled michelia”

#### Type.

CHINA. Yunnan Province: Xichou County, Fadou Mountain, in moist evergreen broad-leaved forest, 1300–1500 m, March 2003, *R.Z. Zhou 0401* (holotype: IBSC n.v.). Same locality, July 2004, *R.Z. Zhou & S.G. Jian 20040701* (paratype: IBSC n.v.).

*Michelia macclurei* Dandy. In: Xia et al. (2008: 85), p.p. quoad syn. *Michelia multitepala* R.Z. Zhou & S.G. Jian.

*Michelia doltsopa* Buch.-Ham. ex DC. In: Sima and Lu (2009: 53) and Sima (2011: 196), both p.p. quoad syn. *Michelia multitepala* R.Z. Zhou & S.G. Jian.

#### Note 1.

There is no data or images held at IBSC for the holotype (Huang Xiangxu, pers. comm., July 2019).

#### Note 2.

The authors of *Michelia multitepala* noted that it closely resembles *M. ingrata* B.L. Chen & S.C.Yang and *M. macclurei* Dandy, but recorded in their comparative diagnosis sufficient morphological differences with these species to substantiate and name *Michelia multitepala* as a distinct new species. *M. multitepala* is recorded as a synonym of *M. doltsopa* Buch.-Ham. ex DC. by the above noted authors. However, in Liu’s classification system of Magnoliaceae (Liu et al. 2004: 381), both *M. macclurei* and *M. ingrata* are placed in Michelia section Anisochlamys Dandy while *M. doltsopa* is placed in Michelia section Michelia.

#### Note 3.

*Michelia multitepala* is sufficiently distinct from *M. doltsopa* (Candolle 1818), to justify its species status, as shown by their known differentiating features compiled in Table 4 below. Additionally, *M. multitepala* is known only to occur at 1300–1500 m on Fadou Mountain in the southeast of Yunnan Province, whereas *M. doltsopa* occurs between 1500–2300 m throughout its widely dispersed geographical area from Yunnan to N Myanmar, NE India, Bhutan and SE Xiyang (Liu et al. 2004: 242), or 2100–2500 m from central Nepal and Burma (Myanmar) to Sichuan and Yunnan (Polunin and Stainton 1999: 19). As a consequence of the substantiation of its specific status, *Michelia multitepala* is here transferred to *Magnolia* in accordance with the past reduction of the remaining genera of subfamily Magnolioideae to the genus *Magnolia*.

**Table 4.** Differentiating features of the species *Michelia multitepala* and *M. doltsopa*.

**Plant feature**	***Michelia multitepala* R.Z. Zhou & S.G. Jian**	***Michelia doltsopa* Buch.-Ham. ex DC**
tree dimensions	15 m tall, 30 cm diameter	30 m tall^†,§^, 1 m diameter
indumentum of leaf buds	rufous appressed-tomentellous	rufous or greyish-white appressed pubescent^§^, orange-rusty hairs on pale green scales^¶^
leaf texture	leathery	thinly leathery^§^
leaf shape and dimensions	elliptic, 14–18 × 5–6.5 cm	elliptic-oblong, 10–22 × 5–7 cm^§^ (10–18(–22) × 3.5–8 cm^††^)
leaf apex	acuminate or short acuminate	short acute or long acute^§^
leaf base	broadly cuneate	obtuse or broadly cuneate^§^
leaf beneath	pale green	pale green and somewhat glaucous beneath^#^, glaucous with orange pubescent veins^¶^
lateral leaf veins	13–15 pairs	10–14 pairs^†^
petiole length and indumentum	1.5–3 cm, rufous appressed tomentellous	1–2 cm^‡‡^, slightly silky grey pubescent^‡^, later glabrescent
stipular scars	none	to ca. 1/5 of petiole length^§^
peduncle indumentum	rufous appressed-tomentellous	densely appressed-villose^§^
tepal number, shape and size	11–12, oblong-lanceolate, 4–6.5 × 0.8–1.7 cm	(8–)12–16, narrowly obovate spoon-shaped 3.6–7.5 × 1.4–3 cm^††^
stamen length	14–16 mm	8–15 mm^††^
gynoecium length	2–2.5 cm	1.5–2 cm^†^
fruit aggregate length	8–15 cm	4–7 cm^§^ (6–10 cm^‡‡^)
shape of carpels	ellipsoid	globose^‡^
flowering period	February–March	March–April^§^

The differentiating features of *Michelia multitepala* are from Jian et al. (2007); those of *Michelia doltsopa* are from Candolle (1818), supplemented by Law et al. (1996: 159)^†^, Lee (1935: 483)^‡^, Liu et al. (2004: 242)^§^, Mitchell and Coombes (1998: 181)^¶^, Polunin and Stainton (1999)^#^, Spongberg (1998: 135)^††^ and Yang et al. (2016: 257)^‡‡^.

### Magnolia pendula

Taxon classificationPlantaeMagnolialesMagnoliaceae

(D.L. Fu) C.B. Callaghan & S.K. Png, comb. nov .

5C77C847-8551-5CBE-8346-228AB6BBB9A0

urn:lsid:ipni.org:names:77209533-1

#### Basionym.

*Yulania pendula* D.L. Fu. In: D.L. Fu et al., Amer. J. Agric. and Forest. 7(5): 220–221, figs. 5 & 6 (2019c).

#### Type.

CHINA. Sichuan Province: Beichuan County, Guixi town, Linfeng village, Yaowang Valley, secondary forest, 1200 m, 2 April 2012, *D. L. Fu 2012040201* (holotype: CAF n.v.). Same locality, 13 September 2012, *D. L. Fu 2012091308* (paratype, CAF n.v.).

#### Chinese name.

垂枝玉兰 meaning “weeping yulan”

#### Note.

The type specimens of *Yulania pendula* cannot be located at the Beijing herbarium of CAF (Wang Hongbin, pers. comm., March 2020).

### Magnolia pilocarpa Z.Z. Zhao & Z.W. Xie var. ellipticifolia

Taxon classificationPlantaeMagnolialesMagnoliaceae

(D.L. Fu, T.B. Zhao & J. Zhao) C.B. Callaghan & S.K. Png comb. nov.

98B703D2-0EEF-5F42-B824-C5717BC53144

urn:lsid:ipni.org:names:77209534-1

#### Basionym.

Yulania pilocarpa (Z.Z. Zhao & Z.W. Xie) D.L. Fu var. ellipticifolia D.L. Fu, T.B. Zhao & J. Zhao. In: D.L. Fu et al., Bull. Bot. Res., Harbin 27(5): 526; figs. 1C–D (2007).

#### Chinese name.

椭圆叶罗田玉兰 meaning “elliptical-leaved Luotian yulan”

#### Type.

CHINA. Henan Province: Xinzheng City, 23 March 2002, *T.B. Zhao et al. 200203231* (holotype: HEAC, flos. n.v.). Same locality, 21 September 2002, *T.B. Zhao et al. 200209211* (paratype: HEAC, folia, ramulus et peruli-alabastrum; n.v.).

*Yulania pilocarpa* (Z.Z. Zhao & Z.W. Xie) D.L. Fu. In: Xia et al. (2008: 76), p.p. quoad syn. Yulania pilocarpa var. ellipticifolia D.L. Fu et al.

Yulania denudata var. pilocarpa (Z.Z. Zhou & Z.W. Xie) Sima & S.G. Lu. In: Sima (2011: 163), p.p. quoad syn. Yulania pilocarpa var. ellipticifolia D.L. Fu et al.

#### Note 1.

The genus *Yulania* Spach (Spach 1839) was resurrected in Flora of China (Xia et al. 2008), but there has not been universal acceptance of this in China, with Yulania again recognised as a subgenus under Magnolia (Ying et al. 2009, Yang et al. 2016).

#### Note 2.

Yulania pilocarpa var. ellipticifolia is sufficiently distinguished from *Y. pilocarpa* to maintain its varietal status by the following features: indumentum of the branchlets (densely pubescent, later glabrous vs. glabrous [Law et al. 2004: 93]); the leaf shape (elliptical, rarely inverted-triangular vs. obovate to broadly obovate [Law et al. 2004]) and the shape and size of the inner 6 tepals (petaloid, 5–7 × 2–3.2 cm vs. nearly spathulate, 7–10 × 3–5 cm [Law et al. 2004]). Additionally, the two taxa are geographically isolated (central Henan Province vs. SE Hubei Province). The illustration of the leaves accompanying the original description of Yulania pilocarpa var. ellipticifolia (Fu et al. 2007: fig.1D) shows them to be in stark contrast to the leaves of *Magnolia pilocarpa* illustrated in Liu et al. (2004: 93).

### Magnolia platypetala

Taxon classificationPlantaeMagnolialesMagnoliaceae

(Hand.-Mazz.) C.B. Callaghan & S.K. Png comb. nov.

62C12D86-680F-54E6-ADB3-39717BAEA48A

urn:lsid:ipni.org:names:77209535-1

#### Basionym.

*Michelia platypetala* Hand.-Mazz. In: Handel-Mazzetti, Anz. Akad. Wiss. Wien, Math.-Naturwiss. Kl. 58(12): 89 (1921).

#### Chinese name.

阔瓣含笑 meaning “broad-petalled (tepalled) michelia”

#### Type.

CHINA. Hunan Province: Yunshan, near Wukang-chow (= Wugang), ca. 950 m, lofty shady forests, April 1919, *Wang Te-Hui (De-Hui Wang) 12281* (p.p. quoad flowering material only, in Handel-Mazzetti, 1921) (holotype: W (possibly destroyed in WWII); isotypes: A online image!, K online image!, SYS!, WU online image!).

Digital images of isotype specimens below accessed 19 March 2019:

isotype [A: 00039059]: http://kiki.huh.harvard.edu/databases/image.php?id=304833

isotype [K: K000681459]: http://apps.kew.org/herbcat/getImage.do?imageBarcode=K000681459

isotype [WU: 0039591]: http://herbarium.univie.ac.at/database/detail.php?ID=71255

*Michelia cavaleriei* Finet & Gagnep. In: Chen and Nooteboom (1993: 1058), Frodin and Govaerts (1996: 55), Wu and Chen (2006: 56), each p.p. quoad syn. *Michelia platypetala* Hand.-Mazz.

Magnolia maudiae var. platypetala (Hand.-Mazz.) Sima (2001: 33).

Magnolia cavaleriei var. platypetala (Hand.-Mazz.) Noot. In: Xia et al. (2008: 49).

Michelia cavaleriei var. platypetala (Hand.-Mazz.) N. H. Xia. In: Xia et al. (2008: 85).

#### Note 1.

Dandy (1928c: 130) provides relevant background information concerning the type collections of *Michelia platypetala* and *M. fallax* from the same general locality in Hunan Province in consecutive years and how they were both initially confused as the former species.

#### Note 2.

As recorded in the synonymy section preceding Note 1, *Michelia platypetala* is noted as a synonym of *M. cavaleriei* and has been made a variety of both *Magnolia maudiae* and *Michelia cavaleriei*, the 2001 and 2008 publications with a noted elevational range of 1200–1500 m despite Handel-Mazzetti’s type collection being made at ca. 950 metres. However, *M. platypetala* retains its species status in Law et al. (1996: 177), Liu et al. (2004: 306), Sima (2011: 219), Deng and Yang (2015: 144) and Yang et al. (2016: 306).

#### Note 3.

Grimshaw and Bayton (2009: 500) record a personal communication received from Richard Figlar in 2007 advising that “this taxon (Magnolia maudiae var. platypetala) probably ought to be recognised at the specific level, as *Magnolia platypetala*, as it differs considerably from *M. maudiae* both in its hairiness and its later bud-break”. Sima (2011: 327), illustrates the contrasting difference between the indumentum of the undersurfaces of the leaves of *M. platypetala* (Plate 3-4H) and that of the leaves of *M. maudiae* (Plate 3-4C). Additionally, in a study by Zhang and Xia (2007) on leaf architecture and its taxonomic significance in respect of subtribe Micheliinae of Magnoliaceae, the pronounced contrast in the leaves of *Michelia platypetala* and *M. cavaleriei* as revealed by stereoscopic magnified imaging (shown at figs. 36 and 37 in their paper), resulted in these authors concluding that these two taxa should be recognised as independent species”. It is apparent that there is now an almost unanimous consensus of the species status of *Michelia platypetala*, which is confirmed by the comparison of its morphological features with those of *M. cavaleriei* compiled in Table 5 below. In view of its distinctive characteristics and accepting the majority recognition by the above-mentioned Chinese authors of *Michelia platypetala* as a genuine species, it is here transferred to *Magnolia* as a consequence of the past reduction of the remaining genera of subfamily Magnolioideae to the genus *Magnolia*.

**Table 5.** Differentiating features of the species *Michelia platypetala* and *M. cavaleriei*.

**Plant feature**	***Michelia platypetala* Hand-Mazz.**	***Michelia cavaleriei* Finet & Gagnep.**
life form	medium-sized tree to 20 m	small-sized tree 7–10 metres
indumentum of branchlets	rufous sericeous	silver-grey or rufous appressed pilose^¶^
indumentum of buds	rufous sericeous	silver-grey or rufous appressed pilose^¶^
leaf shape	oblong or elliptic-oblong	narrowly oblong or narrowly oblanceolate-oblong^¶^
leaf dimensions	11–18(–20) × 4–6(–7) cm (12–17 × 4.5–6.5 cm^†^)	10–20 × 3.5–6.5 cm^¶^ (8–21 × 2.5–5 cm^#^)
leaf apex	acuminate or abruptly narrowed short-acuminate	acuminate or short-acute^¶^
leaf base	broadly cuneate or obtuse	cuneate or broadly cuneate^¶^
leaf indumentum abaxially	greyish-white appressed puberulent or rufous appressed hairs	silver-grey or rufous pilose, appressed when young^¶^
lateral leaf veins	8–14 pairs	11–15 pairs^§^
petiole length	2–3 cm^†^	2 cm (0.7–1.5 cm^#^)
pedicel (peduncle) length	0.5–2 cm	1.5–2.5 cm^§^
bract scar number	2	2–3
tepal number and shape	9 (9–11^†^), obovate-elliptic or elliptic	ca. 12 (10–12^#^): obovate-elliptic^¶^
tepal length (outer 3)	5–7 cm	2.5 cm (2.5–4 cm^¶^)
stamen / anther length	ca. 1 cm / ca. 6 mm	1.2–1.4 cm / ca. 8 mm^§^
gynoecium shape, length and indumentum	cylindric, 6–8 mm, grey or golden puberulent	narrowly ovoid, ca.10 mm, glabrous except for few bristly hairs towards apex of the carpels^‡^
gynophore length	ca. 5 mm	ca. 4 mm^§^
number of ovules	ca. 8 in each immature carpel	2 in each immature carpel
fruit aggregate length	5–15 cm	5–10 cm^¶^
mature carpels shape and size	ellipsoid, rarely globose or ovoid, 1.5–2(–2.5) × 1–1.5 cm	obovoid or ellipsoid, 1.5–2 cm long^§^
flowering period	March–April	March^§^

The distinguishing features of *Michelia platypetala* are mainly cited from Law et al. (1996: 177), Liu et al. (2004: 306) and Yang et al. (2016: 306)^†^, because the description of *M. platypetala* Hand.-Mazz. (1921) includes the composite description of 2 species, including for the fruit of the subsequently named *M. fallax*. The features of *M. cavaleriei* are from Finet and Gagnepain (1906), supplemented by Dandy (1928c: 130)^‡^, Law et al. (1996: 184)^§^, Liu et al. (2004: 229)^¶^ and Yang et al. (2016: 239)^#^.

### Magnolia puberula

Taxon classificationPlantaeMagnolialesMagnoliaceae

(D.L. Fu) C.B. Callaghan & S.K. Png comb. nov.

B16DCB25-85E5-5B4A-AC3D-B97CEA28256D

urn:lsid:ipni.org:names:77209537-1

#### Basionym.

*Yulania puberula* D.L. Fu. In: D.L. Fu et al., Amer. J. Agric. and Forest. 7(5): 208–209, fig. 3 (2019a).

#### Chinese name.

短毛玉兰 meaning “short-haired yulan”

#### Type.

CHINA. Hubei Province, Wudang Mountain, ca. 970 m, 26 March 2018, *D.L. Fu 2018032601* (holotype: CAF, fl. n.v.). Same locality, 8 October 2017, *D.L. Fu 2017100801* (paratype: CAF, fr. n.v.).

#### Note.

The type specimens of *Yulania puberula* cannot be located at the Beijing herbarium of CAF (Wang Hongbin, pers. comm., March 2020).

### Magnolia pubipedunculata

Taxon classificationPlantaeMagnolialesMagnoliaceae

(Q.W. Zeng & X.M. Hu) C.B. Callaghan & S.K. Png comb. nov.

E3D31149-61BA-55B0-A2B9-79B7935429CB

urn:lsid:ipni.org:names:77209538-1

#### Basionym.

*Manglietia pubipedunculata* Q.W. Zeng & X.M. Hu. In: X.M. Hu et al., PloS ONE l4 (3): 4–5, fig. 1 (e0210254: 2019). [13 March 2019 – epublished]

#### Chinese name.

柔毛花梗木莲 meaning “pubescent-peduncled manglietia”

#### Type.

CHINA. Yunnan Province: Wenshan Prefecture, Maguan County, Miechang Town, Daxinzhai Village, Donggualin, Huashikeng, evergreen broad-leaved forests, 1453 m, 104°05'21"E; 22°54'50"N, 14 May 2004, *Q.W. Zeng 89* (holotype: IBSC n.v.). Same locality, 9 September 2003, *Q.W. Zeng 80* (paratype: IBSC n.v.).

#### Note.

There are no data or images held at IBSC for the holotype (Huang Xiangxu, pers. comm., July 2019).

### Magnolia pubipetala

Taxon classificationPlantaeMagnolialesMagnoliaceae

(Q.W. Zeng) C.B. Callaghan & S.K. Png comb. nov.

E7658C72-C0F5-5F42-9741-B974CA3E53A0

urn:lsid:ipni.org:names:77209539-1

#### Basionym.

*Manglietia pubipetala* Q.W. Zeng. In: Q.W. Zeng et al., Pakistan J. Bot.(6): 1917, 1919 + 1918, fig. 1 (2007).

#### Chinese name.

毛瓣木莲 meaning “hairy-tepals manglietia” (this Chinese name is often erroneously applied to *Manglietia rufibarbata* which has glabrous tepals)

#### Type.

CHINA. Yunnan Province: Maguan County, Bazhai, evergreen broad-leaved forests, ca. 1500 m, 14 May 2002, *Ren-zhang Zhou 0256* (holotype: IBSC online image!). Yunnan Province: Xichou County, Fadu, Hemawan, evergreen broad-leaved forests, ca. 1600 m, 2 May 1979, *Gao Ting-xiang & Zhu Dai-qing 05* (paratype: IBSC n.v.). Yunnan Province: Kunming Botanical Garden, introduced 1987 from Yunnan Province’s Malipo County, Jingchang, evergreen broad-leaved forests, 1400 m, 3 May 2003, *Zheng Qing-wen* 67 (paratype: IBSC!).

holotype (IBSC): http://www.docin.com/p-1050989203.html (Sima 2011: 313, photo 2-48).

*Manglietia rufibarbata* Dandy. In: Xia et al. (2008: 60), Sima and Lu (2009: 30) and Sima (2011: 68), each p.p. quoad syn. *Manglietia pubipetala* Q.W. Zeng.

#### Note.

*Manglietia pubipetala* Q.W. Zeng is considered as conspecific with *M. rufibarbata* Dandy by the above authors. However, *M. pubipetala* can be sufficiently differentiated from *M. rufibarbata* Dandy to justify its species status, as shown by the comparative morphological features included in Table 6 on the following page (adapted from Table 1, Zeng et al. 2007). *M. pubipetala* is therefore transferred to *Magnolia* consistent with the past reduction of the remaining genera of subfamily Magnolioideae to the genus *Magnolia*.

**Table 6.** Differentiating features of species *Manglietia pubipetala* and *M. rufibarbata*.

**Plant feature**	***Manglietia pubipetala* Q.W. Zeng**	***Manglietia rufibarbata* Dandy**
indumentum of branchlets	brown villose	densely rufous villose
leaf shape	narrowly obovate-elliptic	oblanceolate or oblanceolate-oblong or obovate-oblong
leaf apex	caudate-acuminate	acuminate or subacuminate
leaf base	cuneate	cuneate or obtuse or occasionally rounded
leaf dimensions	13–17.5 × 4.5–6 cm	10–25 × 4–9 cm^†^
leaf indumentum abaxially	glaucous, densely brown villose	rufous pubescent, especially near midrib
leaf texture	papery	thinly leathery
secondary lateral leaf veins	ca. 10–12 pairs	ca. 12–18 pairs
petiole length / indumentum	1.2–1.5 cm, brown villose	up to 3 cm, rufous villose or tomentose
stipules	brown villose, adnate to petiole	stipules externally densely rufous villose, adnate to petiole only lower 1/3
tepal number	9	11 (9–12^†^)
tepal size (outer 3) and indumentum	3.8–4.0 × 2.5–2.7 cm, pale brown pubescent	ca. 3 × 2 cm^†^ , glabrous^‡^
stamen scars length	6–7 mm	ca. 10–12 mm
gynoecium shape	narrowly obovoid-ellipsoid	ovoid-oblong

The differentiating features of *Manglietia pubipetala* are from Zeng et al. (2007) and those of *M. rufibarbata* are from Dandy (1928), supplemented by Liu et al. (2004: 190)^†^, Zeng et al. (2007)^‡^.

### Magnolia rubriflora

Taxon classificationPlantaeMagnolialesMagnoliaceae

(Y.W. Law & R.Z. Zhou ex F.G. Wang, Q.W. Zeng, R.Z. Zhou & F.W. Xing) C.B. Callaghan & S.K. Png comb. nov.

3EFD0C24-9C04-51FB-9163-F392A616D1DE

urn:lsid:ipni.org:names:77209540-1

#### Basionym.

*Michelia rubriflora* Y.W. Law & R.Z. Zhou ex F.G. Wang et al., Pakistan J. Bot. 37(3): 559, fig. 1 (2005).

#### Chinese name.

红花含笑 meaning “red-flowered michelia”

#### Type.

CHINA. Hainan: Mount Jianfengling, 500–600 m, 31 October 2001, *Zhou Ren-zhang* 0265 (holotype: IBSC n.v.). Guangdong Province: Guangzhou, Magnolia Garden of Guangdong Forest Research Institute, 8 October 2001, *Zhou Ren-zhang 0265b* (paratypes: IBSC!; P online image!).

Digital image of paratype specimen below accessed 19 March 2019:

paratype [P: P00852399]: http://mediaphoto.mnhn.fr/media/1445779250360OrFutLDauT0PI7UU

*Michelia mediocris* Dandy. In: Xia et al. (2008: 85), p.p. quoad syn. *Michelia rubriflora* Y.W. Law & R.Z. Zhou.

#### Note.

While *Michelia rubriflora* is noted as a synonym of *M. mediocris* in Flora of China (Xia et al. 2008), the present authors agree with Wang and co-authors that *Michelia rubriflora* can be more than sufficiently differentiated from *M. mediocris* by the diagnostic features of these two species included in Table 1 of their paper (Wang et al. 2005), to substantiate its species status. A more comprehensive analysis of their differentiating features is compiled in Table 7 below. *Michelia rubriflora* also does not key out with the original validating description for *M. subulifera* (Dandy 1930:212), with which it shares synonymy under *M. mediocris* in Flora of China. Evidently an independent species, *Michelia rubriflora* is transferred in the present paper to the genus *Magnolia* by reason of the past reduction of the remaining genera of subfamily Magnolioideae to that genus.

**Table 7.** Differentiating features of the species *Michelia rubriflora* and *M. mediocris*.

**Plant feature**	***Michelia rubriflora* Y.W. Law & R.Z. Zhou**	***Michelia mediocris* Dandy**
tree dimensions	to 15 m × 25 cm dbh	35 m x 90 cm dbh^†^ (30 m x 190 cm dbh)^‡^
indumentum of buds	greyish-white or pale brown appressed pilose	rufous appressed puberulent^¶^
indumentum of branchlets	greyish-white or pale brown appressed pilose	appressed grey or yellowish-brown tomentose
leaf shape	ovate-elliptic	elliptic or elliptic-oblong
leaf dimensions	5–9 × 2.5–3.5 cm	6–13 × 3–5 cm^§^
leaf indumentum abaxially	greyish-white or pale brown appressed pilose	initially appressed greyish pubescent (greyish-white appressed puberulent)^¶^
leaf texture	leathery	thinly leathery^¶^
lateral leaf veins	9–11 either side of midrib	12–15 either side of midrib
stipular scars	1–2 mm long	none^¶^
petiole length and indumentum	1–2.5 cm, greyish-white or pale brown appressed pilose	1.5–3 cm^§^, initially appressed grey tomentellous, then glabrescent
tepal number /colour	9, red	9–10^#^, white^¶^
tepal size and shape	2.5–3.5 × 1.0–1.2 cm, lanceolate	1.8–2.2 × 0.5–0.8 cm, spathulate^§^
stamen length /colour	1.5–1.7 cm, red	1.0–1.5 cm^§^, yellowish-green
gynophore	not exserted above androecium	extended well above androecium (illustration)^¶^
flowering period	October–November	December–January^¶^ [China] February–March ^#^ [Vietnam]
fruiting period	October–November of the next year	August–September ^¶^ [China] September–October^#^ [Vietnam] of the same year

The differentiating features of *Michelia rubriflora* are from F.G. Wang et al. (2005) and those of *M. mediocris* are from Dandy (1928a), supplemented by Chen and Nooteboom (1993: 1073)^†^, Deng and Yang (2015: 142)^‡^, Law et al. (1996: 180)^§^, Liu et al. (2004: 292)^¶^, Sam et al. (2004)^#^.

### Magnolia rufisyncarpa

Taxon classificationPlantaeMagnolialesMagnoliaceae

(Y.W. Law, R.Z. Zhou & F.G. Wang) C.B. Callaghan & S.K. Png comb. nov.

F5F53223-24AF-510E-8AF7-A7CC31563508

urn:lsid:ipni.org:names:77209541-1

#### Basionym.

*Manglietia rufisyncarpa* Y.W. Law, R.Z. Zhou & F.G. Wang. In: F.G. Wang et al., Nordic J. Bot. 24(5): 519, fig. 1 (2004).

#### Chinese name.

红雌蕊木莲 meaning “red gynoecium manglietia”

#### Type.

CHINA. Yunnan Province: Wenshan, Mount Laojun, 1600 m, 12 May 2001, *Zhou Ren-zhang 008* (holotype IBSC!; isotype: IBSC n.v.). Same locality, 1800 m, 26 April 2001, *Zhou Ren-zhang 0134* (paratype: IBSC n.v.). Guangdong Province: South China Botanical Garden, 30 April 1997, *Zhou Ren-zhang 134* (paratypes: IBSC n.v.; P online image!). Digital image of paratype specimen below accessed 15 March 2020:

paratype [P: P00634914]: http://mediaphoto.mnhn.fr/media/1443127138308WwtO3rNrsfBvSzZP

*Manglietia insignis* (Wall.) Blume. In: Xia et al. (2008: 56), Sima and Lu (2009: 26) and Sima (2011: 102), each p.p. quoad syn. *Manglietia rufisyncarpa* Y.W. Law et al.

*Magnolia insignis* Wall. In: Khuraijam and Goel (2015: 109), p.p. quoad syn. *Manglietia rufisyncarpa* Y.W. Law, R.Z. Zhou & F.G. Wang.

#### Note.

*Manglietia rufisyncarpa* is listed as a synonym of *M. insignis* in Flora of China (Xia et al. 2008), by Sima and Lu (2009) and by Sima (2011). However, the present authors agree with Wang and co-authors that *M. rufisyncarpa* can be more than sufficiently differentiated from *M. insignis* (Wall.) Bl. by the diagnostic characters of these two species compiled by Wang et al. (2004: Table 1), to substantiate its independent species status. Additionally, *M. rufisyncarpa* flowers from April–May whereas *M. insignis* flowers from May–June (Liu et al. 2004: 156). Also, among the many *Manglietia* photos in Magnolias of China, the bright red gynoecium of this species, alluded to in its Chinese name, is particularly noticeable as one of only a few exhibiting this colour, with *M. insignis* displaying a green gynoecium. *Manglietia rufisyncarpa* also does not key out with the original validating descriptions for *M. maguanica* Chang & B.L. Chen, *M. yunnanensis* Hu or *Magnolia shangpaensis* Hu, with which it shares synonymy under *Manglietia insignis* in Flora of China. In view of the above, *M. rufisyncarpa* is transferred in the present paper to *Magnolia*, consistent with the past reduction of the remaining genera of subfamily Magnolioideae to the genus *Magnolia*.

### Magnolia septipetala

Taxon classificationPlantaeMagnolialesMagnoliaceae

(Z.L. Nong) C.B. Callaghan & S.K. Png comb. nov.

885FC7B5-A2CA-50D0-A86C-41BD71B231A0

urn:lsid:ipni.org:names:77209542-1

#### Basionym.

*Michelia septipetala* Z.L. Nong. In: *Guihaia* 13(3): 220–221, fig. 1 (1993).

#### Chinese name.

七瓣含笑 meaning “seven-petals (tepals) michelia” (the tepals in fact are recorded as 7–9)

#### Type.

CHINA. Jiangxi Province: Xinfeng County, Jinpen Shan, in woods, 21 May 1986, *Nong Zhi-lin 086067* (holotype: IBK, fl. white n.v.). Other specimens recorded: Same locality? *Nong Z.L. 086167*. Jiangxi Province: Shangyou County, Wuzhifeng, Guangu Shan, 670 m, 23 November 1976, *Nong Z.L. 760347* (JXAU online images!). Digital images of specimen *760347* with collector noted as Shi Xinghua, accessed 19 March 2019:

[JXAU: 0001182]: http://www.cvh.ac.cn/spm/JXAU/JXAU0001182

[JXAU: 0001183]: http://www.cvh.ac.cn/spm/JXAU/JXAU0001183

[JXAU: 0001184]: http://www.cvh.ac.cn/spm/JXAU/JXAU0001184

*Michelia fujianensis* Q.F. Zheng. In: Xia and Deng (2002: 130) and Xia et al. (2008: 83), both p.p. quoad syn. *Michelia septipetala* Z.L. Nong.

*Michelia caloptila* Y.W. Law & Y.F. Wu. In: Sima (2011: 234), p.p. quoad syn. *Michelia septipetala* Z.L. Nong.

#### Note.

The holotype specimen of *Michelia septipetala* cannot be found at IBK (Xu Wei-bin, pers. comm., July 2019). However, *M. septipetala* can be easily differentiated from both *M. fujianensis* and *M. caloptila*, the 2 species under which it is noted in synonymy above, by the comparison of their morphological and phenological characteristics summarised in Table 8 below.

**Table 8.** Differentiating features of *Michelia septipetala*, *M. fujianensis* and *M. caloptila*.

**Plant feature**	***Michelia septipetala* Z.L. Nong**	***Michelia fujianensis* Q.F. Zheng**	***Michelia caloptila* Y.W. Law & Y.F. Wu**
maximum height	28 m (30 m^†^)	to 16 m^§^	ca. 15 m^¶^
bark colour	greyish-white	greyish-brown	grey
indumentum of buds	densely ferrugineus-tomentose	densely cinnamon-coloured tomentose	brown tomentose
indumentum of branchlets	densely ferrugineus-tomentose	densely cinnamon-coloured tomentose	brown tomentose
leaf shape	oblong-elliptic	oblong or narrowly obovate-elliptic	narrowly elliptic or elliptic
leaf dimensions	8–16 × 2.8–5.5 cm	6–11 × 2.5–4 cm	9–16 × 2.5–5 cm
leaf apex / base	short acuminate / broadly cuneate	acute / rounded	acuminate or caudate-acuminate / cuneate
leaf indumentum adaxially	almost glabrous	densely short-tomentose at midrib	glabrous
leaf indumentum abaxially	ferrugineus-pubescent, denser at midrib	densely ferrugineus or brownish-yellow appressed sericeous	minutely brown tomentose
lateral leaf veins	11–13 pairs	8–9 pairs	7–12 pairs
petiole length / indumentum	5–7 mm, densely ferrugineus pubescent	10–15 mm, densely cinnamon tomentose	5–10 mm, brown tomentose
peduncle indumentum	densely ferrugineus-tomentose	densely cinnamon-coloured tomentose	not known
tepal number and shape	7–9: external 3 tepals obovate, internal tepals narrowly obovate	15–16: spathulate-oblong (12–17, outer 3 tepals narrowly obovate, inner tepals obovate, or narrowly ovate^§^)	6–9: obovate-oblong^#^
stamen number and length	ca. 20, 10–15 mm	number not known, 4–5.5 mm	ca. 35 (photo^#^), length not known
filament length	4–5 mm	1–1.5 mm	not known
gynoecium length	narrowly cylindric, ca. 20 mm	cylindric, ca. 5 mm	not known
gynophore length	ca. 8 mm	ca. 1 mm	not known
immature carpels	ca. 20, densely yellow-brown sericeous, with 2–3 ovules each carpel	pubescent, most aborted	not known
gynophore in fruit	yellow-brown tomentose, 18–25 mm long	pilose, 2–2.5 mm long	ca. 20 mm long
fruit aggregates	7–13 cm long	2–3 cm	4–10 cm long
mature carpels	sessile, oblong or rounded, 1–1.8 × 0.9–1.3 cm with 1–3 seeds	obovoid, 1.5–2 cm × ca. 1.2 cm with 1 seed	broadly ovate or suborbicular, 1–1.8 cm long with 1–4 seeds.
flowering period	May (–June?)	January–February^‡^ December–January^§^	not known
fruiting period	November	October–November^§^	September^¶^

The differentiating features of *Michelia septipetala* are cited from Nong (1993) and Liao and Guo (2010)^†^; those of *Michelia fujianensis* from Zheng (1981)^‡^, supplemented by Liu et al. (2004: 260)^§^, with those of *Michelia caloptila* from Law and Wu (1984) and Liu et al. (2004: 228)^¶^, who each note the flowers as then unknown, plus Yang et al. (2016: 237)^#^.

### Magnolia sinoconifera

Taxon classificationPlantaeMagnolialesMagnoliaceae

(F.N. Wei) C.B. Callaghan & S.K. Png comb. nov.

7317065E-D56E-5FB7-A88F-8CDC7576048E

urn:lsid:ipni.org:names:77209543-1

#### Basionym.

*Manglietia sinoconifera* F.N. Wei. In: *Guihaia* 13(1): 5, fig. *s.n*. (1993).

#### Chinese name.

那坡木莲 meaning “Napo manglietia”

#### Type.

CHINA. Guangxi Zhuang Autonomous Region: Guilin Botanical Garden (cultivated; introduced from Napo County, W Guangxi), 3 June 1991, *Wei Fa-nan 1910* (holotype: IBK n.v.).

*Manglietia dandyi* (Gagnep.) Dandy. In: Xia et al. (2008: 54), p.p. quoad syn. ?*Manglietia sinoconifera* F.N. Wei.

#### Note 1.

The holotype specimen of *Manglietia sinoconifera* cannot be found at IBK (Xu Wei-bin, pers. comm., July 2019).

#### Note 2.

Some of the features distinguishing *Manglietia sinoconifera* from *M. dandyi*, under which it is questionably placed as conspecific in Flora of China due to uncertainty over its status (because the holotype could not be sighted), are listed in Table 9 below. *M. sinoconifera* (to 10 m) also does not key out with the description for the large-leaved *M. megaphylla* Hu & W.C. Cheng (1951), a tree to 40m (Liu et al 2004), with which it shares synonymy under *M. dandyi* in Flora of China. *Manglietia sinoconifera* is recognised as a genuine species in Yang et al. (2016: 213–214), wherein its introduction to Guilin Botanical Garden from Napo County is recorded as 1973 (18 years earlier than stated in the protologue).

**Table 9.** Differentiating features of the species *Manglietia sinoconifera* and *M. dandyi*.

**Plant feature**	***Manglietia sinoconifera* F.N. Wei**	***Manglietia dandyi* (Gagnep.) Dandy**
life form	ca. 10 m	to 15 m^‡^
indumentum of branchlets	densely light reddish-brown	initially soft red pilose, finally ash-grey and almost glabrous
leaf shape	oblanceolate	ovate or broadly lanceolate
leaf dimensions	15–24 × 5.5–8 cm	16–17 × 7–8 cm (16–24 × 5–8.5 cm^‡^)
leaf apex	cuspidate	short acuminate
leaf base	cuneate	obtuse
leaf indumentum abaxially	appressed brown pubescent	red pilose
petiole length and indumentum	2.2–3 cm, appressed brown pubescent	3 cm (1.2–2.3 cm^‡^), red pilose
lateral leaf vein pairs	14–19	8–13^‡^
tepal number and shape	11: outer 3 oblong, inner 8 generally obovate and spathulate	9–11: outer 3 obovate-oblong^‡^, intermediate obovate, innermost oblanceolate
tepal dimensions and indumentum (outer 3)	6.5 × 3.5 cm, glabrous	2–2.2 × 1.5–1.7 cm, pubescent externally at base^‡^
stamen length	10–13 mm	5.5–7 mm^‡^
gynoecium length	ca. 25 mm	10–13 mm^‡^
ovules in each carpel	12	2–10^‡^
flowering period	May^†^	April^‡^

The distinguishing features of *Manglietia sinoconifera* are cited from Wei (1993) supplemented by Yang et al. (2016: 214)^†^ and those of *M. dandyi* from Gagnepain (1939 as *Magnolia dandyi*) supplemented by Chen and Nooteboom (1993: 1037)^‡^.

### Magnolia sonlaensis

Taxon classificationPlantaeMagnolialesMagnoliaceae

(Q.N. Vu) C.B. Callaghan & S.K. Png comb. nov.

6ED0B152-D1A3-57DB-A4C3-33479FCC5C38

urn:lsid:ipni.org:names:77209544-1

#### Basionym.

*Michelia sonlaensis* Q.N. Vu. In: Q.N. Vu et al., Nordic J. Bot. 37(9): 2–3, figs. 1,2 (2019).

**Vietnamese name**: Giổi sơn la, meaning “Son La michelia”

#### Type.

VIETNAM. Son La Province: Yen Chau District, Muong Lum Municipality, Lum village, degraded secondary vegetation, 2270 m, 104°28'44.25"E, 21°00'56.53"N, 1 May 2018, *Nam 152018.2* (holotype: VNF!). Same locality, 2275 m, 104°29'30"E, 21°00'47"N, 2 March 2001, *D.K. Harder et al. 7092* (paratypes: HN!, MO n.v.). Same locality, 2270 m, 104°28'44"E, 21°00'56"N, 19 May 2017, *Nam 1952017* (paratype: VNF n.v.). Same locality, 2272 m, 04°28'44.30"E, 21°00'60"N, 13 April 2019, *Nam 1342019* (paratype: VNF n.v.). Same region, 915 m, 104°28'?"E, 21°00'59"N, 29 December 2010, *Nam 291210.5*; *Nam 291210.6*; *Nam 291210.7* (paratypes: VNF n.v.).

### Magnolia urceolata

Taxon classificationPlantaeMagnolialesMagnoliaceae

(D.L. Fu, B.H. Xiong & X. Chen) C.B. Callaghan & S.K. Png comb. nov.

6B6D0A6B-61BF-5D33-91F3-FF9D705A6896

urn:lsid:ipni.org:names:77209546-1

#### Basionym.

*Yulania urceolata* D.L. Fu, B.H. Xiong & X. Chen. In: D.L. Fu et al., Amer. J. Agric. and Forest. 7(5): 219–220, fig. 4 (2019b).

#### Chinese name.

宽瓣玉兰 meaning “wide-capsuled yulan”

#### Type.

CHINA. Lectotype: Liu Yuhu in Zheng W.J. (Ed) Flora of Trees of China 1: 459; fig. 139 (1983). Guizhou Province: Weining County, 2300 m, 30 September 2017, *D.L. Fu 2017093001* (paratype: CAF, fr. n.v.). *D.L. Fu 2009052401* (paratype: CAF, young fr.). Henan Province: Zhengzhou City (cultivated), *D.L. Fu 2012032001* (paratype: CAF, fl. n.v.).

#### Note.

The type specimens of *Yulania urceolata* cannot be located at the Beijing herbarium of CAF (Wang Hongbin, pers. comm., March 2020).

### Magnolia xinningia

Taxon classificationPlantaeMagnolialesMagnoliaceae

(Y.W. Law & R.Z. Zhou ex Q.X. Ma, Q.W. Zeng, R.Z. Zhou & F.W. Xing) C.B. Callaghan & S.K. Png comb. nov.

2E97DEB1-18B5-5B34-9570-BB259C58AA7B

urn:lsid:ipni.org:names:77209547-1

#### Basionym.

*Michelia xinningia* Y.W. Law & R.Z. Zhou ex Q.X. Ma et al., Pakistan J. Bot. 37(1): 37, fig. 1 (2005).

#### Chinese name.

新宁含笑 meaning “Xinning michelia”

#### Type.

CHINA. Hunan Province: Xinning County, Ziyunshan, in evergreen broad-leaved forests, 1500 m, 20 September 1992, *R.Z. Zhou 197* (holotype: IBSC n.v.; isotype: IBSC n.v.).

Michelia cavaleriei Finet & Gagnep. var. cavaleriei. In: Xia et al. (2008: 84), p.p. quoad syn. *Michelia xinningia* Y.W. Law & R.Z. Zhou.

*Michelia foveolata* Merr. ex Dandy. In: Sima & Lu (2009: 55) and Sima (2011: 216), both p.p. quoad syn. *Michelia xinningia* Y.W. Law & R.Z. Zhou.

#### Note 1.

Digital images of *R.Z. Zhou 197* and *0197* were received from IBSC in 2019, but with the collection dates in April 1988 and April 1996 (Ziyunshan, 800 m) respectively, so probably represent paratypes not mentioned in the 2005 protologue.

#### Note 2.

In Flora of China (Xia et al. 2008), the 9-tepalled *Michelia xinningia* from Hunan, with a published height by the naming authors of 20 m, appears incongruously as a synonym of the ca. 12-tepalled M. cavaleriei var. cavaleriei with a height to 10 m (Liu et al. 2004: 229; Xia et al. 2008: 8; Deng and Yang 2015: 148). This would indicate that this remains about the maximum height of *M. cavaleriei* since being described as a small tree of 4–7 metres more than a century earlier (Finet and Gagnepain 1906: 573), based on a collection from Guizhou ca. 400 km distance from the type locality of *Michelia xinningia* in Hunan. This discrepancy in their heights indicates that *M. xinningia* was evidently meant to appear in Flora of China as a synonym of the then new combination M. cavaleriei var. platypetala (Hand.-Mazz.) N.H. Xia of the same height. However, the present authors agree with the abstract and Latin diagnosis of the authors of *M. xinningia* which indicate it to be sufficiently distinguished from M. cavaleriei var. platypetala (Ma et al. 2005: Table 1), to warrant species status, as has been recognised in Xing et al. (2009: 212) and Yang et al. (2016: 331). Also, *Michelia xinningia* can easily be differentiated from *M. foveolata*, under which it is made a synonym by Sima and Lu (2009) and included as such in Sima (2011: 216), by the comparative features compiled in Table 10.

**Table 10.** Differentiating features of the species *Michelia xinningia* and *M. foveolata*.

**Plant feature**	***Michelia xinningia* Y.W. Law & R.Z. Zhou**	***Michelia foveolata* Merr. ex Dandy**
maximum height	20 m	30+ m
bark colour	greyish-brown	pale grey or dark grey^#^
indumentum of buds	golden villose	densely rufous tomentellous^#^
indumentum of branchlets	golden villose	densely rufous tomentellous^#^
leaf shape	narrowly elliptic	oblong-elliptic, elliptic ovate or broadly lanceolate^#^
leaf dimensions	12–18 × 4.5–5.5 cm	17–23 × 6–11 cm^#^
leaf texture	Leathery	thickly leathery^#^
lateral leaf veins	8–9 pairs	16–20 pairs (16–26 pairs^§^)
leaf abaxially	golden villose with brown pilose midrib	densely coppery-red tomentellous^#^
petiole length and indumentum	1–1.5 cm (1.5–2 cm^†^), golden villose	1.5–4 cm^††^, silky brown pubescent^¶^
tepal number, colour, with shape and size of outer 3	9, white, obovate, 4–5 × ca. 2 cm (7–9 tepals in photo Xing et al. 2009: 213)	9–12, pale yellow with purplish base, broadly ovate, 6–7 cm long#
staminal complex length	ca. 15 mm	ca. 22–25 mm^‡^
stamen number	30–35	ca. 50^§^
filament colour	Red	dark purple^#^
anther length	ca. 0.8 cm	1.5–2 cm^§^
gynoecium length	ca. 1.6 cm	2–3 cm^§^
gynophore length	15–20 mm	12–15 mm^‡^
flowering period	April–May	March–May^#^
elevation and distribution	900–1500 m, Xinning, Hunan^#^	500–1800 m, Guangdong, S Guangxi, SE Guizhou, W Hubei, S Hunan, Jiangxi, SE Yunnan^#^

Footnote: The distinguishing features of *Michelia xinningia* are cited from Ma et al. (2005) and Yang et al. (2016)^†^, with those of *M. foveolata* from Dandy (1928b), supplemented by Chen and Nooteboom (1993: 1066)^‡^, Law et al. (1996: 181)^§^, Lee (1935: 485)^¶^, Liu et al. (2004: 256)^#^ and Yang et al. (2016: 272)^††^.

#### Note 3.

Bearing in mind the above discussion and comparative features, *Michelia xinningia* is an obviously distinct species. Therefore it is here transferred to *Magnolia* due to the past reduction of the previous segregate genera of subfamily Magnolioideae to the genus *Magnolia*.

#### Note 4.

A search of the literature has found that *Michelia xinningia* is in cultivation at 4 Chinese botanical gardens, each in which *M. platypetala* and *M. foveolata* are also cultivated (Callaghan and Png 2019b).

## Supplementary Material

XML Treatment for Magnolia admirabilis

XML Treatment for Magnolia albistaminea

XML Treatment for Magnolia baotaina

XML Treatment for Magnolia caloptila

XML Treatment for Magnolia caudata

XML Treatment for Magnolia fallax

XML Treatment for Magnolia gelida

XML Treatment for Magnolia guangnanica

XML Treatment for Magnolia hunanensis

XML Treatment for Magnolia jinggangshanensis

XML Treatment for Magnolia maguanica

XML Treatment for Magnolia maudiae

XML Treatment for Magnolia multitepala

XML Treatment for Magnolia pendula

XML Treatment for Magnolia pilocarpa Z.Z. Zhao & Z.W. Xie var. ellipticifolia

XML Treatment for Magnolia platypetala

XML Treatment for Magnolia puberula

XML Treatment for Magnolia pubipedunculata

XML Treatment for Magnolia pubipetala

XML Treatment for Magnolia rubriflora

XML Treatment for Magnolia rufisyncarpa

XML Treatment for Magnolia septipetala

XML Treatment for Magnolia sinoconifera

XML Treatment for Magnolia sonlaensis

XML Treatment for Magnolia urceolata

XML Treatment for Magnolia xinningia

## Appendix 1

List of the acronyms of institutional herbaria appearing in this paper.

**A** Arnold Arboretum Herbarium (of Harvard University Herbaria), Cambridge, Massachusetts, USA

**BJFC** Forestry Herbarium, Beijing Forestry University, Xiaozhuang, Beijing, China

**CAF** Dendrological Herbarium, Chinese Academy of Forestry, Haidian, Beijing, China

**HEAC** Henan Agricultural University Herbarium, Zhengzhou, Henan, China

**HFBG** Herbarium, Forestry Botanical Garden of Heilongjiang, Dongliqu, Harbin, Heilongjiang, China

**HFTC** Herbarium, Hunan Forestry Technical College, Hengyang, Hunan, China

**HN** Herbarium, Vietnam Academy of Science and Technology, Hanoi, Vietnam

**IBK** Herbarium, Guangxi Institute of Botany, Yanshan, Guilin, Guangxi, China

**IBSC** Department of Taxonomy Herbarium, South China Institute of Botany, (SCBI) Chinese Academy of Sciences, Wushan, Guangzhou, Guangdong, China

**JXAU** Dendrological Herbarium, Department of Forestry, Jiangxi Agricultural University, Meiling, Nanchang, Jiangxi, China

**K** Royal Botanic Gardens Herbarium, Kew, Surrey, London, UK

**L** Leiden University Branch (Rijksherbarium), National Herbarium of the Netherlands, Leiden, the Netherlands

**LBG** Herbarium, Lushan Botanical Garden, Lushan, Jiangxi, China

**MO** Herbarium, Missouri Botanical Garden, St. Louis, Missouri, USA

**P** Herbarium National de Paris, Muséum National d’Histoire Naturelle, Paris, France

**PE** Laboratory of Systematic and Evolutionary Botany Herbarium, Institute of Botany, Chinese Academy of Sciences, Xiang Shan, Beijing, China

**SIF** Dendrological Herbarium, Forestry School of Sichuan, Dujiangyan, Sichuan, China

**SYS** Biology Department, Botanical Division Herbarium, Zhongshan University (Sun Yat-sen University), Guangzhou, Guangdong, China

**VNF** Vietnam Forestry Herbarium, Hanoi, Vietnam

**W** Herbarium, Natural History Museum, Wien, Austria

**WU** Herbarium, Institute of Botany, University of Vienna, Austria

**ZJFC** Dendrological Herbarium, Department of Forestry, Zhejiang Forestry University, Linan, Zhejiang, China

Herbaria references

The above herbarium acronyms and their institutes were located in the following publications:

Chen SC, Li JL, Zhu XY, Zhang ZY (1990) Bibliography of Chinese Systematic Botany (1949–1990). Guangdong Science & Technology Press, Guangzhou. iv + 810 pp. [In Chinese and English] [Chinese Herbaria, pp. 667–684; Herbarium Abbreviations, pp. 685–698]

Fu LK, Zhang XC, Qin HN, Ma JS (Eds) (1993) Index Herbariorum Sinicorum. China Science and Technology Press, Beijing. vii + 458 pp. [In Chinese and English]

Holmgren PK, Holmgren NH, Barnett LC (Eds) (1990) Index Herbariorum. Part 1. The Herbaria of the World. Eighth Edition. Regnum Vegetabile Vol. 120, New York Botanical Garden (on behalf of the International Association for Plant Taxonomy), The Bronx, New York, x + 693 pp.

Herbaria acronyms may now be searched at: http://sweetgum.nybg.org/science/ih/ [acc: 27.04.2020]

Jin SY, Chen YL (1994) A Catalogue of Type Specimens (Cormophyta) in the Herbaria of China. Science Press, Beijing. xi + 716 pp. [In Chinese] [Magnoliaceae: pp. 453–457; Herbaria acronyms: pp.696–708]
